# Development of a Project-Based Learning Program on High-Risk Newborn Care for Nursing Students and Its Effects: A Quasi-Experimental Study

**DOI:** 10.3390/ijerph19095249

**Published:** 2022-04-26

**Authors:** Hyun-Young Koo, Young-Eun Gu, Bo-Ryeong Lee

**Affiliations:** 1 Research Institute of Nursing Science, College of Nursing, Daegu Catholic University, 33 Duryugongwon-ro 17 gil, Nam-gu, Daegu 42472, Korea; hykoo@cu.ac.kr; 2 Incheon Foundation for Arts and Culture, Department of Culture Management, Graduate School, Inha University, 100 Inha-ro, Michuhol-gu, Incheon 22212, Korea; gracia810@ifac.or.kr

**Keywords:** learning, intensive care unit, neonatal, infant, newborn, students, nursing

## Abstract

Project-based learning (PjBL) allows nursing students to participate in real problem-solving, construct knowledge, and improve their nursing skills in the process of accomplishing meaningful projects. This study was conducted to develop a PjBL program on high-risk newborn care for nursing students and evaluate its effects. A quasi-experimental study using a nonequivalent control group pretest–posttest design was employed between June and December 2021. The participants were 45 nursing students (24 in the experimental group and 21 in the control group). A PjBL program involving the creation of an educational video clip about high-risk newborn care for nursing students was developed, and the experimental group took part in PjBL. The participants’ nursing competency for high-risk newborns, self-leadership, and practicum-related stress were assessed. In the experimental group, nursing competency for high-risk newborns increased and practicum-related stress decreased to a greater extent than in the control group. However, the change in self-leadership was not significantly different between the experimental and control groups. PjBL effectively improved students’ nursing competency for high-risk newborns and decreased their practicum-related stress. PjBL will be utilized to enhance nursing students’ expertise in high-risk newborn care.

## 1. Introduction

Nurses play an essential role in the recovery of high-risk newborns’ health and in the systematic management of neonatal care units. In order to become a competent nurse in the clinical field, nursing students take part in clinical practice to learn the process of assessing newborns’ conditions, quickly identifying health problems, and solving those problems [1].

However, as professional intensive management is emphasized due to the recent increase in high-risk newborn birth [2,3], the need for competent performance of nurses is increasing. Simultaneously, due to the work overload of nurses and legal and ethical issues to protect vulnerable patients, direct nursing practice of students is limited in the clinical field [4,5]. In a neonatal intensive care unit (NICU), nursing students experienced anxiety, fear, and stress due to weak infants (e.g., premature infants or very low birth weight infants) and unfamiliar and complicated medical equipment, prompting them to withdraw to avoid disturbing nurses’ work [6]. In addition, students wanted to be provided with opportunities to hear detailed explanations about nursing and to observe nursing up close in the NICU, while nurses said that it was difficult to guide passive students in a busy situation and wanted students to have more active attitudes [7].

Therefore, various nursing education methods are being implemented to solve difficulties in the education field [8]. It is necessary to help nursing students actively participate in high-risk newborn care practicums and self-directed learning, and PjBL can be applied for these purposes. PjBL is an educational method that enables learners to apply knowledge, improve skills, and complete outcomes by participating in solving authentic problems [9]. PjBL is similar to problem-based learning (PBL) in that it deals with real-world problems and helps students learn by collecting and reconstructing knowledge through student-centered team activities. However, there is a major difference in that PjBL produces clear and visible outcomes [10]. By producing outcomes through PjBL, learners see that their efforts lead to meaningful outcomes and their interest in specialized fields increases [9]. College students who participated in PjBL classes showed high satisfaction and felt pleasure in the participation process [11]. Moreover, the process of achieving goals together allows students to learn proactively to perform their roles and improves their motivations [12]. The process of collaborating with other students during clinical practice provides students an opportunity to learn from each other, build strong bonds, and maintain relationships, which is a necessary virtue of nurses [13]. Nursing students recognized PjBL as a way to improve teamwork and enhance problem-solving and decision-making skills, and an opportunity to fill in lacking experience and knowledge about authentic situations [14].

Thus, this study aimed to develop a PjBL program on high-risk newborn care for nursing students, and to evaluate its effects on nursing competency for high-risk newborns, self-leadership, and practicum-related stress. This study is significant in that it applied PjBL to NICU clinical practice, which is expected to encourage nursing students to engage in active learning during clinical practice while producing meaningful outcomes.

## 2. Methods

### 2.1. Study Design

**Figure 1:**
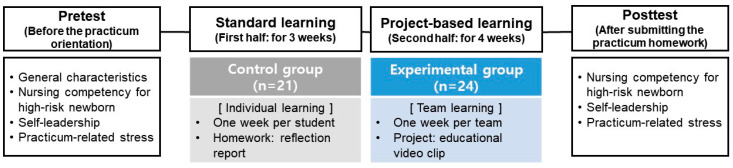
Research design.

A quasi-experimental study using a nonequivalent control group pretest–posttest design was employed at a university in South Korea (Figure 1). This study report followed the Transparent Reporting of Evaluations with Nonrandomized Designs (TREND) reporting guidelines [15].

### 2.2. Participants and Setting

Nursing students at a nursing college in a metropolis of South Korea were recruited through convenience sampling, and third-year students who voluntarily consented in written form to participate in the study were included. The inclusion criteria were students who were in the process of earning 1-credit (45 h) from the NICU practicum. Students who did not consent to participate in the study and did not attend a NICU practicum were excluded. The number of participants was calculated using the G*Power 3.1.9.7 (Düsseldorf, Germany) program by applying repeated-measures analysis of variance (ANOVA) and within–between interactions. Based on previous studies [16], an effect size of 0.25, significance level of 0.05, power of 80%, correlation coefficient of 0.30, and number of measurements of 2 were set, and the calculated number of participants was 23 in each group [17]. Considering a dropout rate of 10%, a total of 50 participants (25 in each group) were recruited. As a result of vacancies due to leave of absence, 24 participants in the experimental group and 21 participants in the control group were finally included in the study, without additional dropouts.

In order to prevent the spread of the experimental effect, the students who practiced in the NICU in the first half of the practicum were assigned to the control group and the students in the second half were assigned to the experimental group.

### 2.3. Development and Evaluation of Project-Based Learning for High-Risk Newborn Care for Nursing Students

The PjBL program on high-risk newborn care for nursing students was developed and evaluated by applying the double-diamond process, a service design method developed by the Design Council [18], consisting of the discovery, definition, development, and delivery stages.

#### 2.3.1. Discovery

The literature on neonatal nursing practicum education and guidance [7,19,20,21] was reviewed. From June to September 2021, surveys and interviews of three clinical practice faculty members (CPFs), three clinical nursing instructors (CNIs), three nursing college graduates, and ten senior students who had experienced NICU practice who consented to participate in the study were conducted.

The following problems in high-risk newborn care practicum education were identified: Nursing students felt anxiety due to premature and fragile high-risk newborns and the complicated medical equipment. They also experienced boredom and helplessness since they could not provide direct nursing care or treatment to protect high-risk newborns, and nursing performance and even observations were restricted to prevent spread of infection.

#### 2.3.2. Definition

The problems in practicum education for high-risk newborn care were determined as nursing students lack the opportunity to learn skills through authentic nursing, and face a sense of helplessness and lack of learning motivation as their activities are restricted during clinical practice.

#### 2.3.3. Development

The PjBL program involving the creation of an educational video about high-risk newborn care for nursing students was developed. The educational themes were the NICU environment and developmentally supportive care, incubator neonatal nursing, ventilator nursing care, and phototherapy nursing care.

On the first day of the practicum, a CPF gave teams a project to produce an educational video clip and explained that the purpose of the video clip was to teach high-risk newborn care to peers who have not participated in the practicum. The CPF assigned teams to one of four themes, provided a worksheet for planning the production of the video clip, and guided each individual team to review the literature on the theme. On the second day, teams set specific learning goals for the video clips through discussion and an additional literature review, identified data to collect, and wrote a production plan. Based on the feedback from the CPF and CNI, the teams reviewed and revised the production plan. As teams performed the project, the teams used in-school practice rooms, NICU space, medical equipment, and models in consultation with the CPF and CNI, and were encouraged to ask the CPF and CNI questions or consult them for advice at any time. The teams produced a 10- to 15-min educational video clip using a mobile phone, edited it after the practice, and submitted the final video clip. The video clips will be used as learning materials for next year’s students (Table 1).

The validity of the composition and content of the project were verified by two CPFs, two CNIs, and two nursing college graduates, and they all responded that the project was appropriate or very appropriate.

#### 2.3.4. Delivery

The PjBL program period was October to December 2021. The data of the control group were collected in the first half and one researcher applied PjBL to the experimental group for a week in the second half. In both the control and experimental groups, the same practice location, practice time, practice guidance time, and practice guidelines were applied according to regulations for operating clinical practice. The control group was provided individual tasks (reflection report on the practice content) instead of a team project, and participants were not told to which group they were assigned. The participants themselves completed the pretest and posttest, which were administered as self-reported online surveys to ensure blinding.

The pretest was conducted two days before the NICU practice for both groups. The posttest was conducted within two days after the NICU practice (after the submission of the team project or individual task). In the online survey, if there was an unanswered question, the participants could not proceed to the next page in order to prevent missing values.

### 2.4. Instruments

All instruments were constructed as self-reported questionnaires and were used after receiving approval from the original authors.

#### 2.4.1. Nursing Competency for High-Risk Newborns

Nursing competency for high-risk newborns was measured using a tool developed by the researcher according to the learning objectives of pediatric nursing [19]. A total of 7 items were measured on a 5-point Likert scale (1: not at all, 5: strongly agree). A higher score was associated with higher nursing competency for high-risk newborns. The Cronbach’s α of internal reliability was 0.94 in the pretest and 0.90 in the posttest.

#### 2.4.2. Self-Leadership

The Korean version of the Revised Self-Leadership Questionnaire (RSLQ), which was developed by Houghton and Neck [22] and translated into Korean by Shin et al. [23], was used for self-leadership. A total of 35 items were measured on a 5-point Likert scale (1: strongly disagree, 5: strongly agree). When four negative items were reverse-coded and summed, the higher score indicated higher self-leadership. The Cronbach’s α was 0.70–0.87 in the study of Shin et al. [23] and 0.91 both in the pretest and posttest of this study.

#### 2.4.3. Practicum-Related Stress

For practicum-related stress, a tool developed by Park and Kim [24] was used. A total of 30 items were measured on a 5-point Likert scale (1: not at all, 5: strongly agree). A higher score was associated with higher practicum-related stress. The Cronbach’s α was 0.90 in Park and Kim’s [24] study, and in this study, it was 0.96 in the pretest and 0.97 in the posttest.

### 2.5. Data Analysis

The data were analyzed using SPSS Statistics version 25.0 (IBM Corp., Armonk, NY, USA). The prior homogeneity between the experimental and control groups was tested using the chi-square test and unpaired *t*-test. Repeated-measures analysis of variance was performed to test differences in outcome variables according to the intervention.

### 2.6. Ethical Considerations

For the ethical protection of participants, this study was conducted after receiving approval (CUIRB-2021-0005) from the Daegu Catholic University Institutional Review Board. The study purpose, methods, and procedure were explained to potential participants, and they were informed that study participation and survey responses did not affect their evaluation or academic grade. They were also informed that there was no disadvantage if they did not want to participate and that they could discontinue participation at any time. They were also informed that confidentiality and anonymity would be maintained and that the collected data would be deleted after the study. Participants were included in the study only after providing written consent to participate in the study voluntarily.

## 3. Results

### 3.1. Homogeneity Testing of Participants’ General Characteristics

There were no significant differences in general characteristics (age, sex, academic performance, health status, satisfaction with school life, satisfaction with friendship, satisfaction with the lectures on newborn care) between the experimental group and the control group. There were also no significant differences in nursing competency for high-risk newborns, self-leadership, and practicum-related stress between the experimental group and the control group (Table 2).

### 3.2. The Effects of the Project-Based Learning Program

Although nursing competency for high-risk newborns was not significantly different between groups (F = 0.15, *p* = 0.703), there were differences between time points (F = 62.24, *p* < 0.001) and a significant interaction between group and time (F = 8.23, *p* = 0.006). Therefore, the pattern of change before and after the intervention was significantly different between the experimental group and the control group.

Self-leadership did not differ between groups (F = 1.23, *p* = 0.273), but there was a difference according to time (F = 25.56, *p* < 0.001) and no interaction between group and time (F = 0.16, *p* = 0.688).

Although practicum-related stress did not show significant differences between groups (F = 0.60, *p* = 0.444) or time points (F = 4.05, *p* = 0.051), there was a significant interaction between group and time (F = 5.84, *p* = 0.020), and the pattern of change before and after the intervention was significantly different between the experimental group and the control group (Table 3).

## 4. Discussion

In this study, nursing competency for high-risk newborns among students who participated in the PjBL program, which involved producing an educational video clip on high-risk newborn care, improved to a greater extent than in the control group. Sufficient knowledge is necessary in order to teach others, and nursing knowledge and skills are improved through planning educational content, performing a rehearsal, and reviewing it [25]. Therefore, it is thought that producing an educational video clip effectively improved students’ nursing competency for high-risk newborns.

As nursing students participated in the PjBL program in teams of 5–7 students, they learned from each other naturally through collecting information together, discussing specific content and methods, and sharing knowledge. Team learning has advantages in improving academic achievement, especially for students with lower academic achievement [12]. Therefore, it is recommended to use team projects to help all students achieve nursing competency above a certain level.

Since the goal of producing the educational video clips was to teach peers about high-risk newborn care, we believe that the students strived to accurately identify the appropriate academic level and achievement level while constructing the video clip with easily understandable content for peers. In previous studies on nursing students [26], peers became learning facilitators in pediatric clinical settings, and students worked together with peers to develop practical skills. Furthermore, since nursing students taught peers instead of patients or the general public, they were less nervous and relaxed and could learn in a supportive environment [25].

Practicum-related stress showed a greater decrease in students who participated in the PjBL program than in the control group in this study. In a clinical setting where the nursing workload has increased and students’ access to patients is restricted, it is becoming difficult for students to receive close guidance from nurses [4,5]. Students restrict their activities or withdraw by themselves so as not to disturb nurses’ work, or passively await nurses’ explanations or guidance [6,7]. This situation increases nursing students’ stress and becomes a factor that makes the practicum education unsatisfactory for them. Since this project producing an education video clip allowed students to use space and medical equipment in the NICU through coordination with the CNI, students were able to talk to nurses naturally and had an opportunity to ask questions on medical equipment. Moreover, the project may have reduced practicum-related stress by decreasing their sense of boredom and helplessness since it provided a task to students who had restricted access to patients in a NICU where medical professionals are busy.

Although PjBL has an advantage of increasing learners’ achievement of knowledge and skills by encouraging learning participation, previous studies also reported burdens for learners, including additional learning [9,27]. Nursing students, who are members of the younger generation, are familiar with media such as YouTube and video clips, are accustomed to shooting video clips using a mobile phone, and have experiences with lectures or clinical skill education utilizing video [28,29]. This may help students feel that producing a video clip was not a substantial source of burden or stress.

However, in this study, the pattern of change in self-leadership did not differ before and after the intervention between the experimental group and the control group. PjBL is characterized as student-centered self-directed learning [10], and the self-regulated learning ability of the experimental group who participated in PjBL in a theory class improved compared to the control group in a study on college students [30]. In this study, even though self-leadership of students who participated in the PjBL significantly improved after the intervention, the control group that performed an individual task also showed an increase in self-leadership, and there was no significant difference in the pattern of change between the two groups. This may be because a reflection report, although it was an individual task, also had a positive effect on self-leadership through self-review and evaluation of the practicum content.

Although the effect of PjBL on self-leadership was not different from that of the individual task, this PjBL program was meaningful in that it significantly increased nursing competency for high-risk newborns, a learning goal that is important for nursing students to reach, and reduced practicum stress. The results of this study show that PjBL contributed to achieving learning goals with less stress. Therefore, it can be applied to improve nursing competency in clinical practice, including the NICU as well as in schools.

A limitation of this study is that it was conducted among nursing students at a single nursing college by convenience sampling, and its results should be generalized with caution. In addition, only one posttest was conducted after the intervention in this study, and a follow-up test was not conducted to verify the long-term effect of PjBL. In the future, it will be necessary to conduct a study to verify the long-term effect of PjBL on characteristics such as students’ learning attitudes and communication methods, as well as nursing competency.

## 5. Conclusions

This study was conducted to develop a PjBL program on high-risk newborn care for nursing students and examine its effects. In this study, a PjBL program, which involved producing an educational video clip about high-risk newborn care for nursing students, was developed. The results revealed that the PjBL program was effective for improving students’ nursing competency for high-risk newborns and decreasing their practicum-related stress. The application of PjBL to clinical practice education contributes to the improvement of nursing competency by encouraging nursing students to engage in active learning while producing meaningful outcomes. Therefore, the application of PjBL can be considered at various clinical practice sites.

## Figures and Tables

**Table 1:** Project-based learning for high-risk newborn care for nursing students.

Phase	Content	Time (Duration)
Orientation	Introduction to project-based learning Presenting a video clip topic - NICU environment and developmentally supportive car - Incubator neonatal nursing - Ventilator nursing care - Phototherapy nursing care - Guidance for using the worksheet to plan video clip production	1st day (30 min)
Pre-learning	Literature review on the video clip topic	1st day
Writing a project plan	Discussion and literature review by team - Brainstorming on detailed content related to educational topic - Selecting content for education - Writing learning goals for the video clip - Identifying the data (e.g., information, photos, videos, etc.) to collect - Exploring and selecting information sources to use - Determining the content and length of the video clip - Assigning roles to team members	2nd day (2–3 h)
Reviewing the project plan	Reviewing and revising the project plan with the CPF and CNI	2nd day (30 min)
Making a video clip	Video clip production	2nd−5th day
Submitting a video clip	Video clip editing and submission	-

CNI: clinical nursing instructor; CPF: clinical practice faculty; NICU: neonatal intensive care unit.

**Table 2:** Homogeneity testing of the general characteristics of the participants and outcome variables of the two groups (*n* = 45).

Variables or Categories	Total (*n* = 45)	Exp. (*n* = 24)	Cont. (*n* = 21)	X^2^/t	*p*
	*n* (%) or M ± SD	*n* (%) or M ± SD	*n* (%) or M ± SD		
Age (year)	21.64 ± 1.86	22.04 ± 2.48	21.19 ± 0.60	1.60	0.122
Sex			
Male	4 (8.9)	1 (4.2)	3 (14.3)	-	0.326 *
Female	41 (91.1)	23 (95.8)	18 (85.7)		
Academic performance (percentile)					
<30	12 (26.7)	7 (29.2)	5 (23.8)	0.16	0.685
>30	33 (73.3)	17 (70.8)	16 (76.2)		
Health status					
Healthy	30 (66.7)	16 (66.7)	14 (66.7)	0.00	>0.999
Unhealthy	15 (33.3)	8 (33.3)	7 (33.3)		
Satisfaction with school life					
Satisfied	26 (57.8)	14 (58.3)	12 (57.1)	0.01	0.936
Unsatisfied	19 (42.2)	10 (41.7)	9 (42.9)		
Satisfaction with friendship					
Satisfied	33 (73.3)	18 (75.0)	15 (71.4)	0.73	0.787
Unsatisfied	12 (26.7)	6 (25.0)	6 (28.6)		
Satisfaction with the lectures on newborn care					
Satisfied	28 (62.2)	17 (70.8)	11 (52.3)	1.62	0.203
Unsatisfied	17 (37.8)	7 (29.2)	10 (47.7)		
Nursing competency for high-risk newborns	24.33 ± 4.42	23.29 ± 4.12	25.52 ± 4.53	1.73	0.091
Self-leadership	122.07 ± 14.80	123.71 ± 16.75	120.19 ± 12.34	0.79	0.433
Practicum-related stress	72.62 ± 19.53	73.54 ± 20.79	71.57 ± 18.43	0.33	0.740

* Fisher’s exact test; Cont.: control group; Exp.: experimental group; *p*, level of significance; t, unpaired T test.

**Table 3:** Nursing competency for high-risk newborns, self-leadership, and practicum-related stress between two groups (*n* = 45).

Variables or Groups	Pretest	Posttest	Source	t or F	*p*
	M ± SD	M ± SD			
Nursing competency for high-risk newborns					
Exp. Cont.	23.29 ± 4.12	30.33 ± 3.12	G	0.15	0.703
	25.52 ± 4.53	28.81 ± 3.23	T	62.24	<0.001
			G × T	8.23	0.006
Self-leadership					
Exp. Cont.	123.71 ± 16.75	135.17 ± 14.64	G	1.23	0.273
	120.19 ± 12.34	129.95 ± 15.31	T	25.56	<0.001
			G × T	0.16	0.688
Practicum-related stress					
Exp. Cont.	73.96 ± 21.16	61.39 ± 17.11	G	0.60	0.444
	71.57 ± 18.43	72.71 ± 27.51	T	4.05	0.051
			G × T	5.84	0.020

Cont.: control group; Exp.: experimental group; G: group; *p*, level of significance; t, unpaired T test; T: time.

## Data Availability

Please contact the corresponding author for data availability.
